# Primary Care Case Management of Febrile Children: Insights From the ePOCT Routine Care Cohort in Dar es Salaam, Tanzania

**DOI:** 10.3389/fped.2021.626386

**Published:** 2021-05-28

**Authors:** Josephine van de Maat, Olga De Santis, Lameck Luwanda, Rainer Tan, Kristina Keitel

**Affiliations:** ^1^Radboudumc, Department of Internal Medicine and Radboudumc Center for Infectious Diseases, Nijmegen, Netherlands; ^2^Erasmus MC – Sophia, Department of General Paediatrics, Rotterdam, Netherlands; ^3^Unisanté – University Center for General Medicine and Public Health, Lausanne, Switzerland; ^4^Ifakara Health Institute, Dar es Salaam, Tanzania; ^5^Swiss Tropical and Public Health Institute, Basel, Switzerland; ^6^Department of Pediatric Emergency Medicine, Department of Pediatrics, University Hospital, Inselspital Bern, University of Bern, Bern, Switzerland

**Keywords:** infectious diseases, low-resource setting, primary care, quality of care, decision-support, pediatrics, fever

## Abstract

**Aim:** To provide insight in the primary health care (PHC) case management of febrile children under-five in Dar es Salaam, and to identify areas for improving quality of care.

**Methods:** We used data from the routine care arm of the ePOCT trial, including children aged 2–59 months who presented with an acute febrile illness to two health centers in Dar es Salaam (2014–2016). The presenting complaint, anthropometrics, vital signs, test results, final diagnosis, and treatment were prospectively collected in all children. We used descriptive statistics to analyze the frequencies of diagnoses, adherence to diagnostics, and prescribed treatments.

**Results:** We included 547 children (47% male, median age 14 months). Most diagnoses were viral: upper respiratory tract infection (60%) and/or gastro-enteritis (18%). Vital signs and anthropometric measurements taken by research staff and urinary testing failed to influence treatment decisions. In total, 518/547 (95%) children received antibiotics, while 119/547 (22%) had an indication for antibiotics based on local guidelines. Antibiotic dosing was frequently out of range. Non-recommended treatments were common (29%), most often cough syrup and vitamins.

**Conclusion:** Our study points to challenges in using diagnostic test results, concerns regarding quality of antibiotic prescriptions, and frequent use of non-evidence-based complementary medicines in PHC in Tanzania. Larger studies on diagnostic and treatments processes in PHC in Tanzania are needed to inform effective solutions to support PHC workers in case management of children.

## Introduction

Child health has improved over the last 20 years in the United Republic of Tanzania. In 2013, the country attained the 4th Millennium Development Goal target—to reduce the under-5 mortality rate by two-thirds from 1990 levels (1). Part of this progress is related to the strengthening of primary health care (PHC) and the implementation of the World Health Organization (WHO) Integrated Management of Childhood Illnesses (IMCI) strategy (2). However, mortality rates in children under-five remain high compared to other settings, and infectious diseases are still a main driver of childhood mortality in low-resource settings (3). Timely and accurate diagnosis and treatment of febrile children in PHC is essential to continue improving health outcomes in this vulnerable patient group. Primary care case management of children with acute febrile illnesses follows the IMCI chart booklet, a set of simple, evidence-based clinical treatment guidelines for settings in which sophisticated diagnostic equipment is not available (4). In addition to IMCI, countries usually have national treatment guidelines in use (5).

Besides the availability of evidence-based guidelines, effective implementation of these guidelines and adherence to their recommendations in clinical practice are crucial, but often poor (6–19). Over the past years, antibiotic over-prescription for children at PHC facilities has become a growing concern (20). This is especially true for febrile children, which represent the vast majority of children presenting to primary care (21). Through a better understanding of diagnostic and care processes, including challenges faced by clinicians, more effective strategies to improve quality of care could be developed. To date, more granular data on the quality of facility-based IMCI care in low-resource settings are still needed (7, 12–14, 22, 23). Most studies on adherence to IMCI have focused on clinical assessments and provider factors (12–14). Fewer, smaller studies focused on the diagnostic and treatment pathways (7, 22).

We provide data from the routine care cohort of the ePOCT study, a randomized controlled trial in primary care in Dar es Salaam, Tanzania. The ePOCT study investigated the impact of a novel electronic decision algorithm (ePOCT) on the management of febrile children, compared to using an electronic version of IMCI (24). It also included a prospective routine care cohort to monitor primary care treatment practices in the study area. The present study aims at describing the case management of febrile children under-five enrolled in this cohort study, and to identify areas for improving quality of care.

## Materials and Methods

### Study Design

This is a prospective cohort study of febrile children in primary care in Dar es Salaam, Tanzania, collected as a routine care cohort in parallel to the ePOCT randomized controlled trial. The main outcome measures were the frequencies of and coherence between presenting complaints, diagnostic tests, provider diagnoses and treatment. In addition, we studied the adherence to treatment recommendations of the IMCI chart booklet and national guidelines.

### Setting

Data were collected in a convenience sample of two neighboring public health centers of the study facilities in the Kindondoni District, Dar es Salaam, Tanzania (Facility A and B). Routine clinicians managing febrile children in the participating health centers were asked to take part in the study. In facility A care was provided by two clinicians (clinician 1 and 2) and in facility B by one clinician (clinician 3). All clinicians were certified clinical officers. Clinical officers undergo a 3 year training in basic and applied medicine, and are responsible for the health care of large dispersed rural populations (25). They treat patients independently, without supervision from medical doctors. All clinicians were trained in IMCI but also used the Tanzanian Standard Treatment Guidelines (4, 26). IMCI includes systematic testing for malaria using a malaria rapid diagnostic test (mRDT). As part of the study, vital sign measurements and mRDT results were provided to the clinician. All essential medicines were made available at the facilities during the study (26).

### Population

This study included children aged 2 to 59 months who presented consecutively with an acute febrile illness to the included health centers. The same inclusion and exclusion criteria as the ePOCT trial were used (briefly: history of fever for <7 days and axillary temperature [T] ≥ 37.5°C, no acute poisoning, or trauma) (24). Written informed consent was obtained from all parents or guardians.

### Data Collection

Data collection took place from December 2014 to February 2016. A trained research assistant saw the patients first, collected data in a predefined case report form on presenting complaints of the child (as reported by the caregiver) and measured basic anthropometrics and vital signs: weight, mid-upper-arm-circumference (MUAC), axillary temperature (T), and respiratory rate (RR). In addition, the research assistant inquired systematically about the presence of cough or difficulty breathing. The research assistant also performed an mRDT in all included children. Other diagnostic tests were ordered at the discretion of the treating clinicians. Test results were recorded on a study form by the research assistant. Clinicians were asked to record their diagnoses and prescribed treatments with dosing. Before discharge of the patient, the research assistant checked whether these diagnoses and treatments corresponded to those noted by the clinician in the patient's chart, and clarified all discrepancies with the clinician. Research staff did not interfere with the provider diagnoses, nor with treatments. Health worker consultations were not observed by study staff. It was ensured that all essential medicines were available during the conduct of the study (26). Additional medicines prescribed by the providers were generally purchased by parents in private pharmacies. Telephone follow-up was performed in all children at day 3, day 7 and day 30 after the visit to the health center. In case children had not recovered at day 7, an additional follow-up was performed at day 14.

### Definitions

For the purpose of this analysis, “recommended treatments per clinical diagnosis” were based on the 2008 IMCI and the Standard Treatment Guidelines of Tanzania from 2013 (4, 26). These guidelines were in use during the data collection of the study. If prescribed treatments were not mentioned in these guidelines, other international guidelines were used to define recommended dosages (27–29). Adherence to mRDT test results was defined as not prescribing antimalarial drugs to a patient when the mRDT result was negative, and treating patients with a positive mRDT result with antimalarial drugs. The spectrum of antibiotics was defined in line with other comparative studies on antibiotic prescription (30–32). The following antibiotics were classified as broad-spectrum: penicillins (including beta-lactamase inhibitors), second- and third-generation cephalosporins, macrolides, aminoglycosides, sulfamethoxazole-trimethoprim, tetracyclines, quinolones and chloramphenicol. Other penicillins (including amoxicillin), first-generation cephalosporins and metronidazole were classified as narrow-spectrum. Since clinicians frequently used diagnoses not contained in the IMCI classifications, we did not attempt re-classification of diagnoses per the IMCI diagnostic classifications. Certain diagnoses were grouped per Supplementary File 1 for data analysis. Positive urine dipstick was defined as the presence of either nitrite or leucocytes.

### Statistical Analysis

We used the following descriptive statistics: frequencies of presenting complaints, clinical diagnoses, diagnostic tests, treatments and dosages were calculated. We tested for differences in provider diagnoses and treatments between clinicians using chi-square tests. We also populated 95% confidence intervals (CI) for proportions to allow comparison between providers. Analyses were performed in Stata version 15.1 and SPSS version 25.

### Patient and Public Involvement

There was no patient or public involvement in the design or conduct of this study, nor in the writing of this manuscript.

### Ethics

The original ePOCT study protocol and related documents were approved by the institutional review boards of the Ifakara Health Institute and the National Institute for Medical Research in Tanzania (NIMRrHQ,R.8a,/trI'VoIl. 789), by the Ethikkommission Beider Basel in Switzerland (EKNZ UBE 15/03), and the Boston Children's Hospital ethical review board. Written informed consent was obtained from all parents or guardians.

## Results

### Population

**Figure 1:**
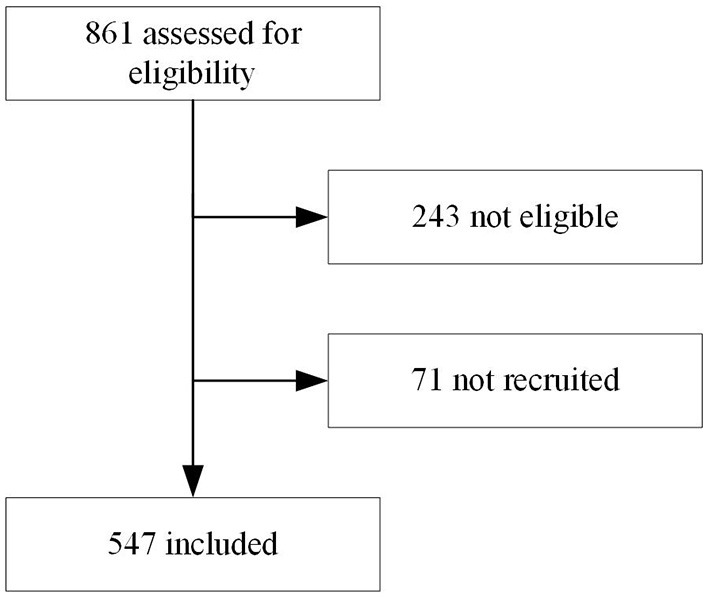
Flowchart of inclusion.

In total 547 children were included (Table 1 and Figure 1), each of the three participating clinicians assessed approximately one third of the population. The population resembled the primary care population in Dar es Salaam of being relatively young (median age 14 month) and presenting mostly with mild disease (7/547 [1%] of children required hospital admission until day 7 follow-up). All children were cured after 2 weeks, but one died at day 20 of follow-up.

**Table 1:** Baseline characteristics.

**Demographics**	***n*/*N* (%) or median (IQR)**
Male sex	259/547 (47%)
Age in months	14 (9–21)
Age group	
−2–11 months	225/547 (41%)
−12–23 months	206/547 (38%)
−24 months and older	116/547 (21%)
Primary care giver	
- Mother	506/514 (98%)
- Father or grandparents	8/514 (2%)
Number of children <18y in household	2 (1–2)
Educational level mother	
- None	30/514 (6%)
- Primary school	348/514 (68%)
- Post primary school	130/514 (25%)
- Post secondary school	6/514 (1%)
Season^a^	
- Rainy	44/547 (8%)
- Post rainy	106/547 (19%)
- Dry	397/547 (73%)
Health center	
- Facility A, clinician 1	149/547 (27%)
- Facility A, clinician 2	234/547 (43%)
- Facility B, clinician 3	164/547 (30%)
Follow-up^b^	
Referral	2/547 (0%)
Admission	7/547 (1%)
Cured at day 3	473/523 (90%)
Cured at day 7	534/544 (98%)
Cured at day 14 (from those not cured at d7)	10/10 (100%)
Died (at day 20)	1/547 (0%)

a *Rainy season in the study area was March to May, post rainy season June to July, dry season August to February*.

b *Not all patients could be reached by telephone during follow-up, resulting in 24 patients with missing follow-up at day 3 and 3 with missing follow-up at day 7*.

### Presenting Complaints and Provider Diagnoses

The most prevalent caregiver-reported presenting complaints other than fever (an inclusion criterion), were cough, or symptoms suggestive of an upper respiratory infection (runny nose, sore throat), see Supplementary File 2. Almost half of the children (249/547, 45%) had two or more presenting complaints besides fever. The most prevalent provider diagnoses were “upper respiratory tract infection (URTI),” “gastroenteritis,” “malaria” and “urinary tract infection (UTI),” as presented in Supplementary File 2. A quarter of the population received two or three diagnoses. Most frequent co-occurring diagnoses were “URTI” and “gastroenteritis” (*n* = 38), “URTI” and “malaria” (*n* = 31), “URTI” and “UTI” (*n* = 17) and “malaria” and “UTI” (*n* = 16), but also many other combinations were present (Table 2). There was significant variability in the five most prevalent diagnoses across clinicians, though differences were not clinically significant besides for malaria (<14% overlap in the 95% CI for all proportions, see below for malaria diagnosis, Supplementary File 3).

**Table 2:** Co-occurring diagnoses.

	**URTI**	**Gastroenteritis**	**Malaria**	**Urinary tract infection**	**Skin infection**	**Vitamin deficiency**
Gastroenteritis	38					
Malaria	31	4				
Urinary tract infection	17	6	16			
Pneumonia	1	0	1	5	0	0
Skin infection	4	2	3	2		
Fever without source	0	0	2	1	1	0
Oral thrush	4	2	0	1	0	0
Vitamin deficiency	3	1	1	4	0	

*URTI, upper respiratory tract infection*.

### Use of and Adherence to Diagnostic Tests

Other than the mRDT (performed by research personnel in all children), diagnostic tests were ordered for 54/547 (10%) of children (Supplementary File 4). Urine dipstick was ordered in 46 cases, but obtained in 31. HIV tests were performed in 2/547 (<1%) of children. An opt-out strategy for HIV testing was in place at the time of the study (i.e., a voluntary HIV test should have been offered to all children presenting for care). Table 3 shows the diagnostic pathways for malaria and UTI. 46/547 (8%) of children were mRDT positive. Average adherence to mRDT results was low (30/46 [65%] of mRDT positive cases were prescribed an antimalarial, Table 3) but this was mainly driven by one clinician, (clinician 1 and 2 together prescribed antimalarials to 28/31 [90%] and 12/13 [92%] of malaria and malaria diagnosis only cases, respectively. Clinician 1 and 2 together prescribed antimalarials to 4/34 [12%] of mRDT negative cases, see Table 3). In total 83 children were diagnosed with “UTI,” of whom 14/83 (17%) underwent urine dipstick testing and 79/83 (95%) were treated with antibiotics. Among the 31 children tested by urine dipstick, the diagnosis of “UTI” was not associated with a positive urine dipstick (either nitrite or leucocytes present) (Fisher's exact test *p* = 0.20). A urine dipstick was ordered for a variety of complaints (Table 3), 8/54 (15%) with a complaint of “fever only” had a urine dipstick ordered (2/31 [7%] of children <24 months). Three of the 4 children diagnosed with “anemia” had a hemoglobin test done, with results <9 g/dL.

**Table 3:** Compliance to recommended diagnostic tests.

**Malaria**		**Urinary tract infection (UTI)**	
**Diagnosis**		**Diagnosis**	
Malaria	85/547 (16%)	UTI	83/547 (15%)
Malaria only	30/547 (5%)	UTI only	33/547 (6%)
**Diagnostic tests**		**Diagnostic tests**	
*All patients routinely tested by mRDT per study procedures independent of main complaint*.		*Main complaints in patients with ordered urine dipstick*	
		Cough	23/46 (50%)
		Upper respiratory infection	10/46 (22%)
		Diarrhea	12/46 (26%)
		Fever only	8/46 (17%)
		Vomiting	7/46 (15%)
		Abdominal pain	6/46 (13%)
		Dysuria	2/46 (4%)
*mRDT in patients with malaria diagnosis (N = 85)*		*Urine dipstick in patients with UTI diagnosis (N = 83)*	
mRDT positive	43/85 (51%)	Urine dipstick ordered	17/83 (20%)
		Urine obtained	14/83 (17%)
		Urine dipstick positive	5/83 (6%)
*mRDT in patients with no malaria diagnosis (N = 459)*		*Urine dipstick in patients without UTI diagnosis (N = 464)*	
mRDT positive	3/459 (1%)	Urine dipstick ordered	29/464 (6%)
		Urine obtained	17/464 (4%)
		Urine dipstick positive	2/464 (1%)
**Treatment**		**Treatment**	
*Recommended: Artemeter-Lumefantrine (Alu)*		*Recommended: antibiotics*	
Alu in malaria diagnoses^a^	29/85 (34%)	Antibiotics in UTI diagnoses	79/83 (95%)
Alu in malaria only diagnoses^a^	12/30 (40%)	Antibiotics in UTI only diagnoses	33/33 (100%)
Alu in mRDT positives^a^	30/46 (65%)	Antibiotics in dipstick positives	7/7 (100%)

a *There was significant variation between the three diagnosis in regards to adherence to mRDT results: Clinician 1 and 2 together prescribed Alu to 28/31 (90%) and 12/13 (92%) of malaria and malaria diagnosis only cases, respectively. Clinician 1 and 2 together prescribed Alu to 4/34 (12%) of mRDT negative cases*.

*mRDT, malaria rapid diagnostic test; UTI, urinary tract infection*.

### Coherence of Clinical Measurements With Diagnoses

Besides axillary temperature (which was an inclusion criterion for the study), weight, MUAC, and RR were measured in all children by trained study staff and made available to the treating clinician. Study staff also specifically inquired about the presence of cough or difficulty breathing. Based on the combination of fever, cough, and elevated respiratory rate (as per IMCI cutoffs), 146/363 (40%) of children with respiratory complaints met the IMCI pneumonia classification; 16/146 (11%) of those received a diagnosis “pneumonia” by the clinician; 120/146 (82%) were diagnosed as “URTI” (Table 4). Fifteen patients (15/547, 3%) had a weight for age <-3 z-score per the WHO growth reference, 1 child also had a MUAC <11.5 cm. One child received a diagnosis of “malnutrition” but this child neither had low weight for age nor low MUAC.

**Table 4:** Provider diagnoses and antibiotics prescribed for patient meeting IMCI criteria for pneumonia.

**Diagnoses**	**Treatment**	**N (%)**
Overall *n* = 146	Any antibiotic	142 (97%)
	**Amoxicillin**	**78 (53%)**
	**Co-Trim^a^**	**7 (5%)**
	Cephalexin	51 (35%)
	IM penicillin	21 (14%)
Pneumonia 16 (11%)	Any antibiotic	16 (100%)
	**Amoxicillin**	**4 (25%)**
	**Co-Trim**	**0 (0%)**
	Cephalexin	10 (63%)
	IM penicillin	5 (31%)
URTI 120 (82%)	Any antibiotic	117 (98%)
	**Amoxicillin**	**70 (60%)**
	**Co-Trim**	**7 (6%)**
	Cephalexin	38 (32%)
	IM penicillin	10 (8%)
Malaria 21 (14%)	Any antibiotic	21 (100%)
	**Amoxicillin**	**10 (48%)**
	**Co-Trim**	**0 (0%)**
	Cephalexin	8 (38%)
	IM penicillin	14 (67%)
Gastroenteritis 15 (10%)	Any antibiotic	14 (93%)
	**Amoxicillin**	**8 (53%)**
	**Co-Trim**	**6 (40%)**
	Cephalexin	3 (20%)
	IM penicillin	1 (7%)
UTI 13 (9%)	Any antibiotic	12 (92%)
	**Amoxicillin**	**6 (46%)**
	**Co-Trim**	**0 (0%)**
	Cephalexin	4 (30%)
	IM penicillin	2 (15%)

*First-line antibiotics indicated per IMCI or the national treatment guidelines are bolded*.

a *Co-Trim, combination of sulfamethoxazole and trimethoprim*.

*IMCI, integrated management of childhood illness; URTI, upper respiratory tract infection; UTI, urinary tract infection; IM, intramuscular*.

### Antibiotic Treatment

Ninety-five percent of the children received an antibiotic treatment (518/547, Table 5A). Amoxicillin was prescribed most frequently (282/518, 54%), followed by cephalexin (134/518, 26%) (Table 5A). Of all children receiving antibiotics, 90/518 (17%) received broad-spectrum antibiotics. 182/547 children (33%) received broad-spectrum or multiple antibiotics. Injectable antibiotics were prescribed in 103/547 (19%) of patients, of which only two had a severe diagnosis indicating the need for intramuscular pre-referral antibiotic treatment. Based on the diagnoses of the clinician, 119/547 (22%) of children had an indication for antibiotic treatment based on IMCI or Tanzanian national guidelines (Supplementary File 5). At least one indicated antibiotic per IMCI or national guidelines was prescribed in 17/119 (14%) of children with an indication for antibiotic treatment (Supplementary File 5). “Appropriateness” did not vary significantly by clinician (chi square *p* = 0.40).

**Table 5:** Treatments prescribed.

**(A) Antibiotics prescribed in all children, and per diagnosis**					
**Antibiotics**	**All children** ***n*** **(%)**	**URTI** ***n*** **(%)**	**Gastroenteritis** ***n*** **(%)**	**UTI** ***n*** **(%)**	**Pneumonia^a^** ***n*** **(%)**
	***N*** **=** **547**	***N*** **=** **327**	***N*** **=** **100**	***N*** **=** **83**	***N*** **=** **31**
Any antibiotic	518 (95%)	310 (95%)	96 (96%)	79 (95%)	31 (100%)
Amoxicillin	282 (56%)	196 (60%)	25 (25%)	42 (51%)	**11 (35%)**
Penicillin	75 (14%)	22 (7%)	3 (3%)	13 (16%)	14 (45%)
Amoxicillin/clavulanic acid	6 (1%)	1 (0%)	0 (0%)	**3 (4%)**	2 (6%)
Amoxicillin/cloxacillin	30 (6%)	16 (5%)	0 (0%)	6 (8%)	2 (6%)
Cephalexin	134 (25%)	86 (26%)	11 (11%)	22 (26%)	18 (58%)
Ceftriaxone	1 (0%)	0 (0%)	0 (0%)	0 (0%)	1 (3%)
Azithromycin	6 (1%)	3 (1%)	2 (2%)	3 (4%)	0 (0%)
Erythromycin	16 (3%)	3 (1%)	12 (12%)	1 (1%)	0 (0%)
Ciprofloxacin	6 (1%)	0 (0%)	4 (4%)	2 (2%)	0 (0%)
Co-trim	55 (10%)	20 (6%)	51 (51%)	5 (6%)	**0 (0%)**
Chloramphenicol	1 (0%)	0 (0%)	0 (0%)	0 (0%)	1 (3%)
Gentamicin	2 (0%)	0 (0%)	0 (0%)	0 (0%)	1 (3%)
Metronidazole	4 (1%)	1 (0%)	3 (3%)	0 (0%)	0 (0%)
**(B) Other treatments prescribed in all children**					
**Other treatments**	**All children** ***n*** **(%)**				
	***N*** **=** **547**				
Cough syrup	95 (17%)				
ORS	85 (16%)				
Vitamins	60 (11%)				
Zinc	60 (11%)				
Artemether-lumefantrine	31 (6%)				
Ibuprofen	22 (4%)				
Skin treatment	16 (3%)				
Domperidone	13 (2%)				
Salbutamol per os	8 (1%)				
Salbutamol inhaler	8 (1%)				
Antihistamine	7 (1%)				
Eye treatment	3 (1%)				
Safe remedies	2 (0%)				
Bella donna	2 (0%)				

*Antibiotic treatment with more than 10% of patients are shaded in gray. Antibiotics indicated per IMCI or the national treatment guidelines are bolded*.

a *non-severe pneumonia*.

*IMCI, integrated management of childhood illness; URTI, upper respiratory tract infection; UTI, urinary tract infection; ORS, oral rehydration solution*.

A first-line recommended antibiotic treatment was prescribed in 11/31 (36%) of children with a “pneumonia” diagnosis. 82/146 (56%) of children with an IMCI pneumonia classification based on the research staff measurements were prescribed a first-line antibiotic treatment (Table 4). Among those with a “UTI” diagnosis, most children (42/83, 51%) received amoxicillin, whereas 3/83 (4%) received the first-line recommended treatment amoxicillin/clavulanic acid. Children who were only diagnosed with “gastroenteritis” (*n* = 51) were prescribed antibiotics in 92% of cases, although these infections are mostly viral. Details of prescribed antibiotics for the most frequent infections can be found in Table 5A.

For prescribed antibiotics, dosing errors were frequent (Table 6). The prescribed dose for amoxicillin was too low in about one-third of prescriptions based on guidelines in use. Out-of-range prescribing was not significantly different among clinicians (chi square *p* = 0.78 for dosing within WHO 2008 range, data not shown). All six recorded azithromycin prescriptions were too high (range 17–71 mg/kg/day). For cephalexin all prescriptions except one were within the range of 25–100 mg/kg/day of the WHO Model Prescribing Information (27). Co-Trim was in 93% of cases prescribed within the WHO 2008 range; 47% of the erythromycin dosages were too low.

**Table 6:** Dosage of antibiotics.

**Recommended daily dose**	**Amoxicillin**	**Azithromycin**	**Cephalexin**	**Ciprofloxacin**	**Co-Trim^a^**	**Erythromycin**
	***N* = 272**	***N* = 6**	***N* = 133**	***N* = 6**	***N* = 54**	***N* = 15**
Dose mg/kg, median (IQR)	44 (38–54)	26 (17–45)	41 (36–47)	31 (23–37)	9 (8–10)	43 (38–54)
WHO 2014 guideline^b^						
Dose within range	86/272 (32%)			0/6 (0%)		
*Dose too high*	0/272 (0%)			1/6 (17%)		
*Dose too low*	186/272 (68%)			5/5 (83%)		
WHO 2008^c^						
Dose within range	191/272 (70%)			0/6 (0%)	50/54 (93%)	
*Dose too high*	4/272 (1%)			1/6 (17%)	0/54 (0%)	
*Dose too low*	77/272 (28%)			5/5 (83%)	4/54 (7%)	
Tanzania guideline^d^						
Dose within range	126/272 (46%)					
*Dose too high*	48/272 (18%)					
*Dose too low*	98 (36%)					
International guideline^e^						
Dose within range	174/272 (64%)	0/6 (0%)	132/133 (99%)	5/5 (83%)	17/54 (31%)	7/15 (47%)
*Dose too high*	0/272 (0%)	6/6 (100%)	0/133 (0%)	1/6 (17%)	32/54 (59%)	1/15 (6%)
*Dose too low*	98/272 (36%)	0/6 (0%)	1/133 (1%)	0/6 (0%)	5/54 (9%)	7/15 (47%)

a *trimetoprim/sulfometoxazole in pediatric tablet 20/100 mg, only the trimethoprim dose is mentioned in the table*.

b *based on WHO 2014 chart booklet (amoxicillin: 4–10 kg = 500 mg/d; 10–14 kg = 1,000 mg/d; 14–19 kg = 1,500 mg/d. Ciprofloxacin <6months 250 mg/d, 6–59 months 500 mg/d, or 30 mg/kg/d)*.

c *based on WHO 2008 chart booklet (amoxicillin: 4–10 kg = 375 mg/d, 10–19 kg = 750 mg/d; Co-Trim: 4–10 kg = 80 mg/d, 10–19 kg = 120 mg/d)*.

d *based on Tanzanian guideline: for pneumonia, recommended dose is 25 mg/kg every 12 h; 20% dosage variation (40–60 mg/kg/d) was assumed*.

e *Pneumonia amoxicillin: 40–120 mg/kg/d based on WHO CAP (community-acquired pneumonia) evidence update 2016 (28). Rest based on WHO Model Prescribing Information (27): azithromycin 10 mg/kg/d with 10% dose variation; Cephalexin: 25–100 mg/kg/d; Ciprofloxacin: 20–40 mg/kg/d; Co-Trim 8 mg/kg/d with 10% dose variation; Erythromycin 40–60 mg/kg/d*.

*WHO, World Health Organization; IQR, interquartile range*.

### Other Treatments

Besides antibiotics, most frequently prescribed medications were paracetamol, cough syrup, oral rehydration solution (ORS), vitamins, and zinc (Table 5B). Recommended treatments according to guidelines at the time of the study are displayed in Supplementary File 6. Prescriptions of those prevalent medications showed some variation between the three clinicians, but not clinically significant (Supplementary File 3). Zinc was prescribed in 45/100 (45%), and ORS in 74/100 (74%) of “gastroenteritis” diagnoses. Cough syrup, which is not indicated per IMCI but can be considered in children with cough per the Tanzanian National Treatment Guidelines, was given to 82/336 (24%) of children with cough. Salbutamol was given to 9/547 (1%) of patients; 7 of whom received it both per os and per inhalator. Treatments not recommended in any guideline included vitamins in 60/547 (11%), domperidone in 13/547 (2%), antihistamines in 7/547 (1%), and bella donna in 2/547 (1%) of patients. When including cough syrup as a non-recommended treatment, 161/547 (29%) of children received a non-recommended treatment.

## Discussion

### Summary of Main Findings

In this descriptive analysis of primary diagnostic and treatment practices for febrile children in Dar es Salaam, Tanzania, we found a very high prevalence of antibiotic prescription. Several issues around antibiotic prescription were identified, including a mismatch between diagnoses and antibiotic treatment, and inaccurate dosing. Misdiagnoses and incorrect treatment prescriptions were common despite provision of respiratory rates, weight and MUAC to clinicians. Adherence to mRDT test results was highly variable. Urine dipstick testing did not appear to change the diagnostic or treatment process in this cohort. Prescription of complementary, non-indicated treatments was frequent.

### Strengths and Limitations

Our study has obvious limitations. The cohort was small and included only three clinicians in two health centers. The generalizability of the findings is hence very limited. However, we did not note relevant provider variability across most domains observed, besides in the treatment of malaria. Dar es Salaam presents a very specific, urban care setting in Tanzania and our findings cannot be generalized to other parts of the country. Our study did not collect data on the consultation process, but rather only on the “inputs” and “outputs” of the consultation. We were hence limited in our ability to explain our findings. At the same time, the lack of observation of the consultation process is also a strength of our study: previous studies in Tanzania often used observations of consultations (13, 14, 22), raising concern for a Hawthorne effect. Even though a Hawthorne effect cannot be excluded in our study, we believe it is limited in the absence of direct observation. Our study also removed barriers that have been previously reported to challenge quality of primary care, such as the availability of diagnostics and medicines, and the lack of time to perform anthropometric and RR measurements (33–35). This allowed us to explore the contribution of such factors to the diagnostic and treatment process. We also provide more granular data on medicine prescriptions in primary care in Tanzania.

### Interpretation and Comparison to the Literature

Overall, our findings point to several challenges faced by clinicians in managing febrile children, which warrant further investigation.

The distribution of the infectious diagnoses (with 68% of diagnoses being likely viral, like URTI and gastroenteritis) reflects recent epidemiological evidence of the study area (36, 37). The high number of viral diagnoses is a change from a recent study in the same area, where under-classification of viral illnesses was found (22). UTI appeared to be over-diagnosed in our cohort [15% of patients vs. an estimated prevalence of around 3–5% reported by similar studies (36, 37)]. Other bacterial infections such as pneumonia and ear infections seemed to be under-diagnosed, although most often covered by antibiotics for other diagnoses.

Interestingly, clinicians hardly used IMCI classifications for their diagnoses, but rather used diagnoses such as “upper respiratory tract infection” and “gastroenteritis” found in national guidelines and standard pediatric textbooks. Especially as Tanzania is moving toward a national electronic medical record system, it will be important to understand how diagnoses are used by primary care clinicians.

Two diagnoses, malaria, and UTI, should have been based on diagnostic testing. For malaria, many studies have raised concerns about the lack of testing and adherence to test results (38–41). We also found concerns regarding adherence to mRDT test results, but the data was driven by a single provider. Urine dipstick results seemed not to influence treatment decisions in our study. Urine dipstick is not included within IMCI, but is in the Tanzanian Standard Treatment Guidelines.

Previous studies pointed to the lack of diagnostic tests as a main challenge in fever management. Our findings underline the need for clear guidance and training in the use and interpretation of diagnostic tests. In addition, a variety of “know-do” gaps have been described to explain non-adherence to IMCI guidelines and diagnostic tests, these include a preference on relying on personal experience, provider priorities, goodness of fit in routine practices as well as intrinsic and extrinsic motivation (12, 14, 42). In this context, it is also interesting that systematically provided RR and anthropometric measurements did not appear to be taken into consideration by clinicians. Even though this systematic measurement was a minor intervention in routine care, it did not seem to change clinical practice. Previous studies have stipulated that a lack of RR and anthropometric measurements are the main barrier to correct classification of IMCI pneumonia and malnutrition cases, which was clearly not the case in our study (33–35).

Our findings confirm the concern about high-volume antibiotic prescription in primary care in Tanzania (20, 37, 43–45). The almost systematic prescription of antibiotics for viral infections (e.g., primarily gastroenteritis and URTI) questions the motivations behind such non-indicated use. Previous studies have reported many underlying factors to explain inappropriate antibiotic prescription. These include pressure from families, discomfort with the possibility that the child could worsen before returning to care, the perception that bacterial illnesses are much more common, understaffing, inability to differentiate causes of illness based on history and physical examination alone, discomfort with diagnostic uncertainty, and insufficient knowledge in managing non-malarial fever in children (12, 23, 40, 46). These findings from other studies may also be a reason for the high volume of medicine prescription in general. Additional diagnostic testing can improve certainty about bacterial or viral etiology of febrile illness and could thereby improve antibiotic prescription. Diagnostic test results should be readily available at the point of care, where treatment decisions are made (47). Bacterial culture or parasite microscopy are unlikely to impact antibiotic prescription in the primary care setting given the complexity to perform, long duration to obtain results, and overall inaccessibility. Rapid viral or antigen tests are usually not available in Sub-Saharan primary care. Other point-of-care tests like c-reactive protein (CRP) have shown to improve antibiotic prescription in the primary care setting in Vietnam (48). In high-income settings, the impact on antibiotic prescription has been variable (49–51). The combination of point-of-care tests and clinical information seems to be most important to improve antibiotic prescription and should be further investigated in the Sub-Saharan primary care setting (24, 48).

Our study raises additional concerns about the quality of antibiotic prescription. Only a small minority of diagnosis-antibiotic prescription pairs aligned with guidance from IMCI or national guidelines. Similarly, a study from Zanzibar reported that only 22% of antibiotic prescriptions were appropriate for the diagnosis (37). Broad-spectrum antibiotics were commonly prescribed and dosing errors were frequent, fueling antimicrobial resistance. Despite the availability of all essential medicines at the facilities during the study, providers opted for second-line agents and non-indicated antibiotics. Our data indicate that health workers would benefit from increased support in picking the correct weight-based dosing. Our findings point to possible avenues that would have to be addressed in a primary care antibiotic stewardship programs.

Another consequence of prescribing non-essential medicines is the additional cost-incurred by parents. One-third of patients were prescribed additional non-evidence-based treatments such as vitamins and cough syrup, putting additional financial burden on families and incurring potential safety risks in children (e.g., domperidone and bella donna).

## Conclusion

The observations from this descriptive study and its limitations again point to the complexity of quality of care in pediatric primary care in Africa. Current evidence in Tanzania is largely limited to small-scale, fragmented reports or focusing on hospital settings or malaria case-management specifically (52, 53). In addition to previous evidence, our report points to challenges in using diagnostic tests other than mRDT, concerns regarding the quality of antibiotic prescriptions, and the frequent prescription of non-evidence based complementary medicines. Future efforts should focus on larger, systematic studies, which include a variety of methods to assess the quality of primary care diagnostic and treatment processes (54, 55). Such methods should combine systematic monitoring with more granular data generation to explain mechanisms for change. In the meantime, effective solutions to support primary health care workers in case management of children are urgently needed.

## Data Availability Statement

The raw data supporting the conclusions of this article will be made available by the authors, without undue reservation.

## Ethics Statement

The studies involving human participants were reviewed and approved by the institutional review boards of the Ifakara Health Institute and the National Institute for Medical Research in Tanzania (NIMRrHQ,R.8a,/trI'VoIl. 789), the Ethikkommission Beider Basel in Switzerland (EKNZ UBE 15/03), and the Boston Children's Hospital ethical review board. Written informed consent to participate in this study was provided by the participants' legal guardian/next of kin.

## Author Contributions

JM was responsible for the design of the paper and the data analysis and was the main author of the draft and revised manuscript. OD contributed to data analysis and writing and revision of the manuscript. RT and LL contributed to the writing and revision of the manuscript. KK initiated the study, was responsible for funding, designed the trial and supervised the data collection and the writing of the paper. All authors substantially contributed to the writing (i.e., drafting and/or critical revision) of the manuscript.

## Collaborators

- John Masimba, Ifakara Health Institute, Dar es salaam, Tanzania.
- Josephine Samaka, Ifakara Health Institute, Dar es salaam, Tanzania.
- Zamzam Said, Ifakara Health Institute, Dar es salaam, Tanzania.
- Hosiana Temba, Ifakara Health Institute, Dar es salaam, Tanzania.
- Frank Kagoro, Ifakara Health Institute, Dar es salaam, Tanzania.
- Valérie D'Acremont, Unisanté – University Center for General Medicine and Public Health, Lausanne, Switzerland; Swiss Tropical and Public Health Institute, Basel, Switzerland.

## Conflict of Interest

The authors declare that the research was conducted in the absence of any commercial or financial relationships that could be construed as a potential conflict of interest.

## Abbreviations

CRP: c-reactive protein
ePOCT: study investigating the impact of a novel electronic decision algorithm
IMCI: Integrated Management of Childhood Illnesses
mRDT: malaria rapid diagnostic test
MUAC: mid-upper-arm-circumference
ORS: rehydration solution
PHC: primary health care
RR: respiratory rate
T: temperature
URTI: respiratory tract infection
UTI: urinary tract infection
WHO: World Health Organization.
