# Supplementary material for: Primary Care Case Management of Febrile Children: Insights From the ePOCT Routine Care Cohort in Dar es Salaam, Tanzania
Source: Front Pediatr. 2021 May 28;9:626386. doi: 10.3389/fped.2021.626386 (PMC8192830; doi:10.3389/fped.2021.626386)
Supplement: Supplementary file 1 [file Data_Sheet_1.PDF]

Supplementary file 1. Diagnostic categories used for analysis and corresponding diagnoses recorded by clinicians.

**Diagnostic category**	**Corresponding diagnoses recorded by clinicians (in decreasing frequency)**
Severe disease	Meningitis, severe pneumonia
URTI	Upper respiratory infection, flu, cold, tonsillitis, sinusitis, gingivitis
Pneumonia	Pneumonia
Ear problem	Otitis media
Gastroenteritis	Gastroenteritis, diarrhea, gastritis, dysentery
Malaria	Malaria
Fever without source	Fever, bacterial infection, blood infection, hyperexia of unknown origin, undefined illness, undefined symptoms
Viral infection	Viral disease, viral infection
Urinary tract infection	Urinary tract infection
Skin infection	Skin infection, folliculitis, skin allergy, tinea capitis,
Abscess	Boils
Malnutrition	Malnutrition
Anemia	Anemia
Vitamin deficiency	Vitamin deficiency, avitaminosis
Oral thrush	Oral thrush, oral candidiasis
Eye problem	Conjunctivitis, eye infection
Intestinal parasites	Intestinal parasites, worms

IMCI classifications (based on 2008 and 2014 chart booklet) are displayed in blue.

Supplementary file 2. Presenting complaints and provider diagnoses

Presenting complaints		Provider diagnosis	
Fever	528/547 (97%)	Upper respiratory tract infection	327/547 (60%)
Fever only	54/547 (10%)	Gastroenteritis	100/547 (18%)
Cough	323/547 (59%)	Malaria	85/547 (16%)
Upper respiratory symptoms (runny nose, sore throat)	159/547 (29%)	Urinary tract infection	83/547 (15%)
Diarrhea	107/547 (20%)	Pneumonia	31/547 (6%)
Vomiting	63/547 (12%)	Skin infection	18/547 (3%)
Abdominal pain	26/547 (5%)	Fever without source	13/547 (2%)
Loss of appetite	23/547 (4%)	Oral thrush	9/547 (2%)
Skin problem	23/547 (4%)	Vitamin deficiency	9/547 (2%)
Mouth/throat problem	14/547 (3%)	Intestinal parasites	7/547 (1%)
Difficulty breathing	12/547 (2%)	Abscess	6/547 (1%)
Dysuria	4/547 (1%)	Anemia	4/547 (1%)
Eye problem	4/547 (1%)	Eye problem	4/547 (1%)
Skin abscess	3/547 (1%)	Severe infection (severe pneumonia/meningitis)	2/547 (0%)
Bloody diarrhea	2/547 (0%)	Ear problem	1/547 (0%)
Convulsion, 1 episode	1/547 (0%)	Viral illness	1/547 (0%)
Ear problem	1/547 (0%)	Other (gingivitis)	1/547 (0%)
No presenting complaint recorded	9/547 (2%)	No diagnosis	2/547 (0%)

Supplementary file 3. Variation in diagnoses and treatment across clinicians

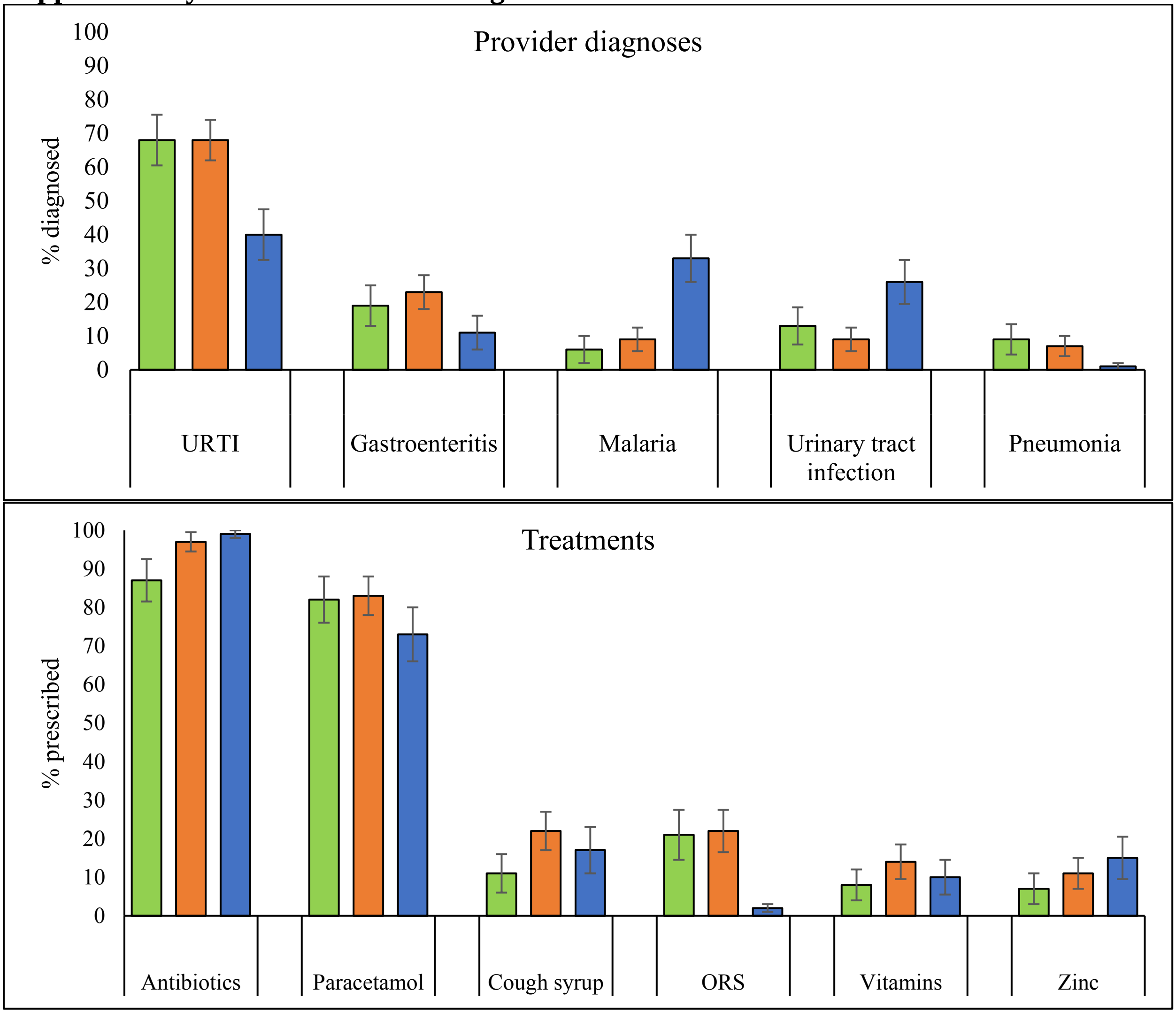

Footnote: Error bars are 95% confidence intervals of % diagnosed or prescribed.
Green = clinician 1, red = clinician 2 (facility A), blue = clinician 3 (facility B).
URTI = upper respiratory tract infection; ORS = oral rehydration solution.

Supplementary file 4. Diagnostic tests ordered

Test	n (% of total number of children)	Test result
Urine dipstick (ordered)	46 (8%)	See table 3
Hemoglobin	4 (1%)	Median 8.9 g/dL (IQR 8.7 – 9.5)
HIV	2 (0%)	Both negative
Stool study	2 (0%)	1 hook work ova; 1 not reported
Blood glucose	2 (0%)	4.9 and 6.7 mmol/L
X-ray	1 (0%)	Soft tissue injury
Sputum aspirate	1 (0%)	Negative

Supplementary file 5. Definition of appropriate antibiotic treatment based on guidelines in use at the time of the study

Diagnostic category	Indicated antibiotic treatments
Meningitis	Ampicillin+gentamicin, ampicillin OR benzyl penicillin + chloramphenicol, ceftriaxone
Severe pneumonia	Amoxicillin/ampicillin+gentamicin; Benzyl Penicillin; chloramphenicol
Pneumonia	Amoxicillin, co-trim
Otitis media	amoxicillin
Dysentery	Metronidazole, ciprofloxacin
Urinary tract infection	Amoxicillin/clavulanic acid
Skin abscess	Flucloxacillin, erythromycin, cephalexin
Folliculitis	Amoxicillin/clavulanic acid; cephalexin

Footnote: Based on WHO 2008 IMCI chart booklet, Tanzania 2013 Standard Treatment Guidelines

Supplementary file 6. Definition of indicated other systemic treatment based on guidelines in use at the time of the study

	Indicated diagnoses	
**Treatment**	**2008 IMCI chart booklet**	**Tanzania 2013 Standard Treatment Guidelines**
Paracetamol	Fever	Fever, pain
Cough syrup	-	Cough (may be considered)
ORS	Dehydration	Dehydration
Vitamins	-	Hook worm infection
Zinc	Diarrhea	Diarrhea
Artemether-lumefantrine	Malaria	Malaria
Ibuprofen	-	Fever
Domperidone	-	-
Salbutamol	Wheezing	Wheezing
Antihistamine	-	Eczema, allergic rhinitis, urticaria

Supplementary file 7. STROBE Statement—Checklist of items that should be included in reports of *cohort studies*

	Item No	Recommendation	Page No
**Title and abstract**	1	(*a*) Indicate the study's design with a commonly used term in the title or the abstract	1
		(*b*) Provide in the abstract an informative and balanced summary of what was done and what was found	2
**Introduction**			
Background/rationale	2	Explain the scientific background and rationale for the investigation being reported	3
Objectives	3	State specific objectives, including any prespecified hypotheses	3
**Methods**			
Study design	4	Present key elements of study design early in the paper	3
Setting	5	Describe the setting, locations, and relevant dates, including periods of recruitment, exposure, follow-up, and data collection	4
Participants	6	(*a*) Give the eligibility criteria, and the sources and methods of selection of participants. Describe methods of follow-up	4
		(*b*) For matched studies, give matching criteria and number of exposed and unexposed	NA
Variables	7	Clearly define all outcomes, exposures, predictors, potential confounders, and effect modifiers. Give diagnostic criteria, if applicable	4
Data sources/ measurement	8*	For each variable of interest, give sources of data and details of methods of assessment (measurement). Describe comparability of assessment methods if there is more than one group	4
Bias	9	Describe any efforts to address potential sources of bias	4
Study size	10	Explain how the study size was arrived at	4
Quantitative variables	11	Explain how quantitative variables were handled in the analyses. If applicable, describe which groupings were chosen and why	5
Statistical methods	12	(*a*) Describe all statistical methods, including those used to control for confounding	5
		(*b*) Describe any methods used to examine subgroups and interactions	
		(*c*) Explain how missing data were addressed	
		(*d*) If applicable, explain how loss to follow-up was addressed	
		(*e*) Describe any sensitivity analyses	
**Results**			
Participants	13*	(a) Report numbers of individuals at each stage of study—eg numbers potentially eligible, examined for eligibility, confirmed eligible, included in the study, completing follow-up, and analysed	5
		(b) Give reasons for non-participation at each stage	
		(c) Consider use of a flow diagram	
Descriptive data	14*	(a) Give characteristics of study participants (eg demographic, clinical, social) and information on exposures and potential confounders	5, T1
		(b) Indicate number of participants with missing data for each variable of interest	
		(c) Summarise follow-up time (eg, average and total amount)	
Outcome data	15*	Report numbers of outcome events or summary measures over time	6

Main results	16	(*a*) Give unadjusted estimates and, if applicable, confounder-adjusted estimates and their precision (eg, 95% confidence interval). Make clear which confounders were adjusted for and why they were included<br>(*b*) Report category boundaries when continuous variables were categorized<br>(*c*) If relevant, consider translating estimates of relative risk into absolute risk for a meaningful time period	NA
Other analyses	17	Report other analyses done—eg analyses of subgroups and interactions, and sensitivity analyses	NA
**Discussion**			
Key results	18	Summarise key results with reference to study objectives	7
Limitations	19	Discuss limitations of the study, taking into account sources of potential bias or imprecision. Discuss both direction and magnitude of any potential bias	8
Interpretation	20	Give a cautious overall interpretation of results considering objectives, limitations, multiplicity of analyses, results from similar studies, and other relevant evidence	8
Generalisability	21	Discuss the generalisability (external validity) of the study results	8
**Other information**			
Funding	22	Give the source of funding and the role of the funders for the present study and, if applicable, for the original study on which the present article is based	11

*Give information separately for exposed and unexposed groups.

Note: An Explanation and Elaboration article discusses each checklist item and gives methodological background and published examples of transparent reporting. The STROBE checklist is best used in conjunction with this article (freely available on the Web sites of PLoS Medicine at http://www.plosmedicine.org/, Annals of Internal Medicine at http://www.annals.org/, and Epidemiology at http://www.epidem.com/). Information on the STROBE Initiative is available at http://www.strobe-statement.org.
